# Expression of cytokine and apoptosis-related genes in bovine peripheral blood mononuclear cells stimulated with *Brucella abortus* recombinant proteins

**DOI:** 10.1186/s13567-016-0311-7

**Published:** 2016-02-11

**Authors:** Young Bin Im, Myunghwan Jung, Min-Kyoung Shin, Suk Kim, Han Sang Yoo

**Affiliations:** Department of Infectious Diseases, College of Veterinary Medicine, Seoul National University, Seoul, 08826 South Korea; College of Veterinary Medicine, Gyeongsang National University, Jinju, 52828 South Korea; Institute of Green-Bio Science and Technology, Seoul National University, Pyeongchang, 25354 South Korea

## Abstract

Brucellosis is a clinically and economically important disease. Therefore, eradication programs of the disease have been implemented in several countries. One hurdle in these programs is the detection of infected animals at the early stage. Although the protein antigens as diagnostic antigens have recently received attention, the exact mechanisms at the beginning of immune responses are not yet known. Therefore, genes encoding five *B. abortus* cellular proteins were cloned and the expressed recombinant proteins were purified. The expression of several cytokine genes (IL-1β, IL-4, IL-6, IL-12p40, IFN-γ, TNF-α, and iNOS) was analyzed in bovine peripheral blood mononuclear cells (bPBMC) after stimulation with the recombinant proteins. Three apoptosis-related genes, Bax, Bcl-2, and TLR4, were also included in the analysis to find out the adverse effects of the proteins to the cells. Each protein induced different patterns of cytokine expression depending on the stimulation time and antigen dose. Expression of IL-6, IL-12p40, and IFN-γ was induced with all of the proteins while IL-1β, IL-4, TNF-α, and iNOS gene expression was not. Expression of apoptosis-related genes was not altered except TLR4. These results suggest that the cellular antigens of *B. abortus* induce both humoral and cellular immunity via the production of IL-6, IL-12p40, and IFN-γ in bPBMC without exerting any adverse effects on the cells.

## Introduction

Brucellosis is a highly contagious zoonosis caused by Gram-negative bacteria of the genus *Brucella.* This disease affects livestock, wild animals, and humans. Ten species of the genus *Brucella* have been identified based on antigen variation and primary hosts [1, 2]. Brucellosis causes significant economic losses not only because it affects animal production (reduced milk production, abortion, delayed conception, and impaired fertility) but also because detection of the disease in a region or country causes enactment of international veterinary regulations as well as restrictions on animal movements and trade [1, 3]. In addition, brucellosis in human can be severely debilitating and remains an important public health concern [1, 4].

Most serological diagnostic methods for detecting *Brucella* infection use antibodies against common *Brucella* antigens [5]. O-polysaccharide (OPS), a well-known immunodominant epitope in smooth lipopolysaccharide (SLPS), is commonly used in serological tests for diagnosing brucellosis [6–9]. Recently, several cellular proteins of *B. abortus* have been considered new diagnostic antigens because traditional diagnostic methods using *Brucella* LPS have low specificity due to cross-reactivity with other relevant bacteria such as *Yersinia enterocolitica* O:9 [10, 11].

*Brucella abortus* is a facultative intracellular bacterial pathogen that can survive intracellular defenses and hamper the induction of host humoral immune responses [12]. These properties help preventing the serological diagnosis of *B. abortus* infection. Infection with *B. abortus* potently activates both the innate and adaptive immune system, leading to a proinflammatory response that favors the T-helper 1 (Th1) responses [13, 14]. Although both antibody- and cell-mediated immune responses can influence the course of *Brucella* infection, the latter is primarily responsible for the clearance of intracellular bacteria [15].

*Brucella* spp. have mechanism that prevent activation of the host innate immune system [16]. Invasion through the digestive tract does not elicit any inflammatory response including cytokine production from the host [17]. Therefore, *Brucella* spp. invade silently or unnoticed by the innate immune system of the host [18].

Cytokines are important for responses to infection. Much attention has thus been given to research on cytokine-mediated inflammatory reactions in cases of brucellosis. Previous studies have revealed that *B. abortus* can induce the production of proinflammatory cytokines such as tumor necrosis factor-α (TNF-α), interleukin (IL)-6, IL-12 and IL-1β by a variety of cell types and in mice [19–23].

Understanding immune mechanisms is an important step for the development of new control measures including diagnostic antigen(s) since most of the antigens have been selected based on reaction with antibodies without considering the immune responses in the host [24–27]. However, the precious mechanisms of *B. abortus* infection including the possible apoptotic activities have not been revealed in the expression of cytokines and apoptosis-related genes, yet. Therefore, to understand the mechanism underlying the immune responses to *B. abortus*, bovine peripheral blood mononuclear cells (bPBMC) were treated with five well-known immunoreactive *B. abortus* cellular proteins. Cytokine production and the expression of genes associated with apoptosis were then analyzed as the first step of understanding in the induction of immune responses.

## Materials and methods

### Cloning and expression of *B. abortus* genes

Genes encoding outer membrane protein 28 (OMP28), malate dehydrogenase (mdh), elongation factor Ts (tsf), arginase (rocF), and metal-dependent hydrolase (0628) of *B. abortus* 544 were amplified by PCR (Table 1), cloned, and expressed with a cold shock expression vector (pCold^TM^ TF DNA; Takara, Japan) in *E. coli* DH5α. Sequences of the primers used for this procedure are shown in Table 1. Nucleotide sequences of the genes were confirmed by sequencing using an automatic sequencer and dye-termination sequencing system.

**Table 1:** **Primer sequences for cloning** ***Brucella abortus*** **genes**

Gene	Primer sequence (5′–3′)	Annealing temperature (°C)	Product size (bp)
Outer membrane protein 28	F: GATCGGATCCAACACTCGTGCTAGCAATTTT	63	753
	R: GATCAAGCTTTTACTTGATTTCAAAAACGAC		
Malate dehydrogenase	F: AATTCGGATCCATGGCACGCAACAAGATT	63	963
	R: AGGCGTCGACTTATTTCAGCGACGGAGC		
Elongation factor	F: AATTCGAATTCATGAGCATTTCCGCATCT	63	918
	R: AGGCCTGCAGTCAGCCCTTGGCGGCTGCGGC		
Arginase	F: AGCGCGGATCCATGCATTGTAAGATTCTG	63	918
	R: AGGCCTGCAGTCAATAGCTGATGGTCGG		
Metal-dependent hydrolase	F: AGCGCGGATCCATGCATTGTAAGATTCTG	63	711
	R: AGCGCTGCAGTTAAGCTTGGAAGCTGTG		

### Purification of the recombinant proteins

Five *E. coli* clones were cultured at 37 °C overnight in 100 mL of LB broth (Duchefa Biochemie, The Netherlands) with 10 μg of ampicillin (Duchefa Biochemie, The Netherlands). Sixty milli liters of the cultures were used to inoculate 1 L of LB broth containing 100 μg of ampicillin. After culturing with shaking at 220 rpm for 3 h at 37 °C, isopropyl β-*D*-1-thiogalactopyranoside (IPTG; Amresco, USA) was added at a final concentration of 0.5 mM and the culture was further incubated overnight at 37 °C in a shaking incubator (Vision Science Co. Ltd., Korea) at 220 rpm. After incubation, the bacterial cells were harvested by centrifugation at 4400 *g* for 20 min. The resulting pellets were resuspended in 40 mL of binding buffer (20 mM Tris–HCl, 8 M urea, 500 mM NaCl, 20 mM imidazole (Sigma, USA) [pH 8.0], and 1 mM β-mercaptoethanol (Sigma, USA) and sonicated at 10 000 Hz in ice water (60% pulse, 20 s pulse/50 s steps, 15 cycles). Supernatants were collected after centrifugation at 4400 × *g* for 20 min. Recombinant proteins were collected using a His SpinTrap (GE Healthcare, UK) according to the manufacturer’s protocol. Concentration of the purified recombinant proteins was measured using a BCA kit (Bio-Rad, USA). The recombinant proteins were analyzed by SDS-PAGE and Western blotting with an anti-His antibody (April Bio Co. Ltd., Korea). LPS contamination in the purified proteins was confirmed by endotoxin assay kit (Toxin Sensor^TM^ Chromogenic LAL endotoxin Assay Kit, GenScript).

### bPBMC isolation and culturing

Blood was collected into conical tubes (Nunc, USA) containing heparin from the jugular vein of clinically healthy Korean native cattle. The cattle were naïve to *B. abortus* exposure. The blood was overlaid onto 15 mL of Histopaque 1077 (Sigma, USA) in a 50 mL conical tube and centrifuged 400 × *g* for 30 min. Next, bPBMC were collected and washed three times with RPMI 1640 medium (Gibco, USA) containing 10% fetal bovine serum (Gibco, USA). The bPBMC concentration was adjusted to 5 × 10^6^ cells/mL and the cells were cultured in a 6-well plate with RPMI 1640 medium containing 10% FBS for 8 h at 37 °C in a 5% CO_2_ atmosphere. After incubation, the bPBMC were stimulated with 5 or 10 μg/mL of the five different recombinant proteins for the indicated time intervals in the figures. LPS was removed by treatment with polymyxin B (10 μg/mL) before stimulation with the proteins. Concanavalin A (ConA, 1 μg/mL) was used as a positive control. All animal procedures were carried out according to the guidelines of the Institutional Animal Care and Use Committee (IACUC) of the Animal and Plant Quarantine Agency (South Korea). The study protocol was approved by the Seoul National University Institutional Animal Care and Use Committee (SNUIACUC: SNU-130916-3).

### Purification of total RNA from bPBMC

Total RNA was isolated from the bPBMC using an RNeasy mini kit (Qiagen, Germany) according to the manufacturer’s protocol. Before reverse transcription of the RNA using a QuantiTect^®^ Reverse Transcription Kit (Qiagen, Germany) was performed, genomic DNA was eliminated with 2 μL of gDNA wipeout buffer (7×), 11 μL of RNase-free water, and 1 μg of template RNA. After incubating the mixture at 42 °C for 2 min, reverse transcription was carried out with 1 μL of quantiscript reverse transcriptase, 4 μL of quantiscript RT buffer (5×), 1 μL of reverse transcription primer mix, and 14 μL of cDNA generated by incubation at 42 °C for 15 min. Quantiscript reverse transcriptase was inactivated by incubation at 95 °C for 15 min. The reaction products were used to analyze gene expression with real-time PCR.

### Analysis of cytokine and apoptosis-associated genes by real-time PCR

Real-time PCR was carried out using the cDNA products after completing reverse transcription-PCR. A Rotor-Gene SYBR Green PCR Kit (Qiagen, Germany) was used with a two-step cycling protocol including denaturation at 95 °C and a combined annealing/extension step dependent upon the primer T_m_ value according to the manufacturer’s protocol. The reaction mixture contained 10 μL of 2× Rotor-Gene SYBR Green PCR Master Mix, 2 μL of the primers, 2 μL of template DNA or cDNA, and 6 μL of RNase-free water. The real-time PCR conditions were as follows: initial denaturation at 95 °C for 5 min followed by 50 cycles of 95 °C for 20 s and annealing at 55 °C or 60 °C for 10 s. The expression of each gene was normalized relative to the expression of β-actin. Genes encoding inducible nitric oxide synthase (iNOS), IL-1β, IL-4, Il-6, IL-12p40, interferon (IFN)-γ, and TNF-α were analyzed. Three apoptosis-related genes, Bcl-2-associated X protein (Bax), B cell lymphoma 2 (Bcl-2), and Toll-like receptor-4 (TLR4), were also analyzed. Sequences of the primers and probes used in this real-time PCR are presented in Table 2.

**Table 2:** **Primer sets used for real-time PCR**

Gene	Primer sequence (5′–3′)	Annealing temperature (°C)	Product size (bp)	Reference
iNOS	F: AGCGGAGTGACTTTCCAAGA	55	97	[40]
	R: TTTTGGGGTTCATGATGGAT			
IL-1β	F: ACCTTCATTGCCCAGGTTTCT	55	120	[40]
	R: TGTTTGGGGTCATCAGCCTCAA			
IL-4	F: CAAAGAACACAACTAAGAAG	55	181	[40]
	R: AGGTCTTTCAGCGTACTTGT			
IL-6	F: TCCAGAATGAGTATGAGG	55	236	[40]
	R: CATCCGAATAGCTCTCAG			
IL-12p40	F: AACCTGCAACTGAGACCATT	55	186	[40]
	R: ATCCTTGTGGCATGTGACTT			
IFN-γ	F: ATAACCAGGTCATTCAAAGG	55	218	[40]
	R: ATTCTGACTTCTCTTCCGCT			
TNF-α	F: TAACAAGCCAGTAGCCCACG	55	277	[40]
	R: GCAAGGGCTCTTGATGGCAGA			
Bax	F: TCTCCCCGAGAGGTCTTTTT	55	151	[41]
	R: TGATGGTCCTGATCAACTCG			
Bcl-2	F: ATGTGTGTGGAGAGCGTCAA	55	146	[41]
	R: CTAGGGCCATACAGCTCCAC			
TLR4	F: TGACATCTTCACAGAACTGACTA	55	164	This study
	R: GGAGTGGTTCATAAAGAAATGTA			
β-actin	F: CGCACCACTGGCATTGTCAT	60	227	[42]
	R: TCCAAGGCGACGTAGCAGAG			

### Statistical analysis

Data are reported as the mean ± standard error of the mean (SEM.) of three or more independent experiments. Statistically significant was determined by the Student *t* test using statistical package for social science (SPSS) software version 21. Differences were considered to be significant if a *p* value was <0.05.

## Results

**Figure 1:**
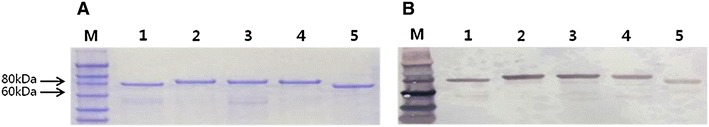
**Analysis of the purified recombinant proteins.** SDS-PAGE (**A**) and Western blotting (**B**) with an anti-His antibody. Lane M: molecular weight markers (Life Technologies, USA), lane 1: outer membrane protein 28, lane 2: malate dehydrogenase, lane 3: elongation factor, lane 4: arginase, lane 5: metal-dependent hydrolase.

Genes encoding five different cellular proteins of *B. abortus* (OMP28, mdh, tsf, rocF, and 0628) were cloned, sequenced, and expressed in *E. coli*. The expressed proteins were purified and analyzed by SDS-PAGE and Western blotting using an anti-His antibody (Figure 1). Generally, treatment with each recombinant protein induced different patterns of cytokine expression depending on stimulation time and dose. However, the expression of apoptosis-related genes was not greatly affected by stimulation with the recombinant proteins.

**Figure 2:**
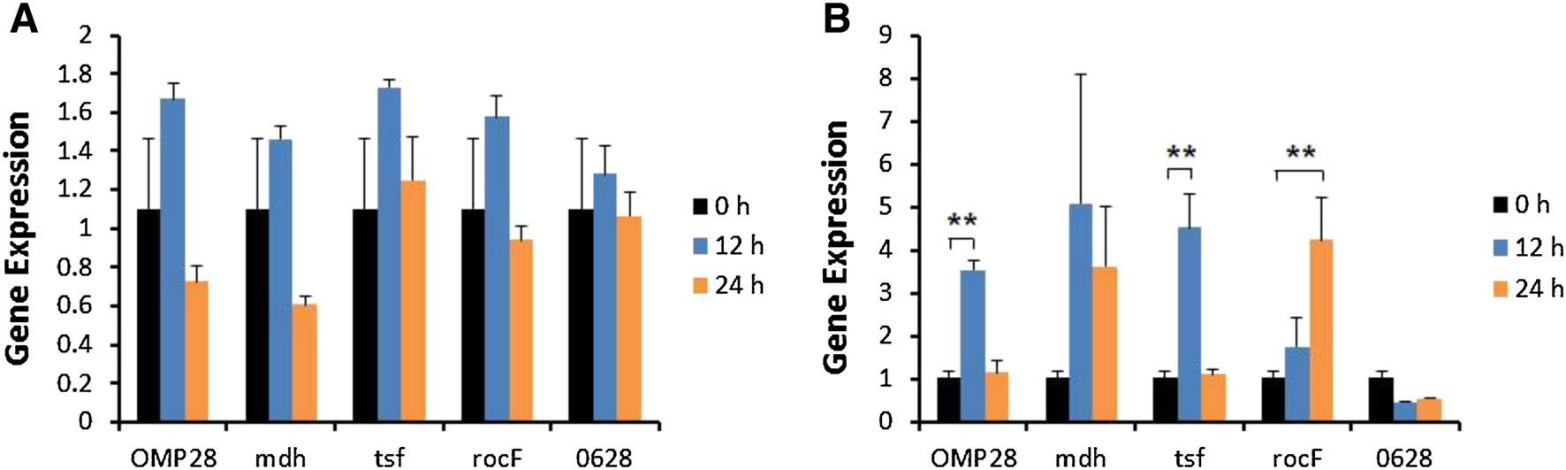
**Gene expression of inducible nitric oxide synthease (iNOS) in bovine PBMC.** Bovine PBMC were stimulated with 5 μg (**A**) and 10 μg (**B**) of five different recombinant proteins (OMP28, mdh, tsf, rocF, and 0628) of *Brucella abortus* at 0, 12, and 24 h. Gene expression was analyzed by real-time quantitative RT-PCR and normalized by the expression of β-actin.

**Figure 3:**
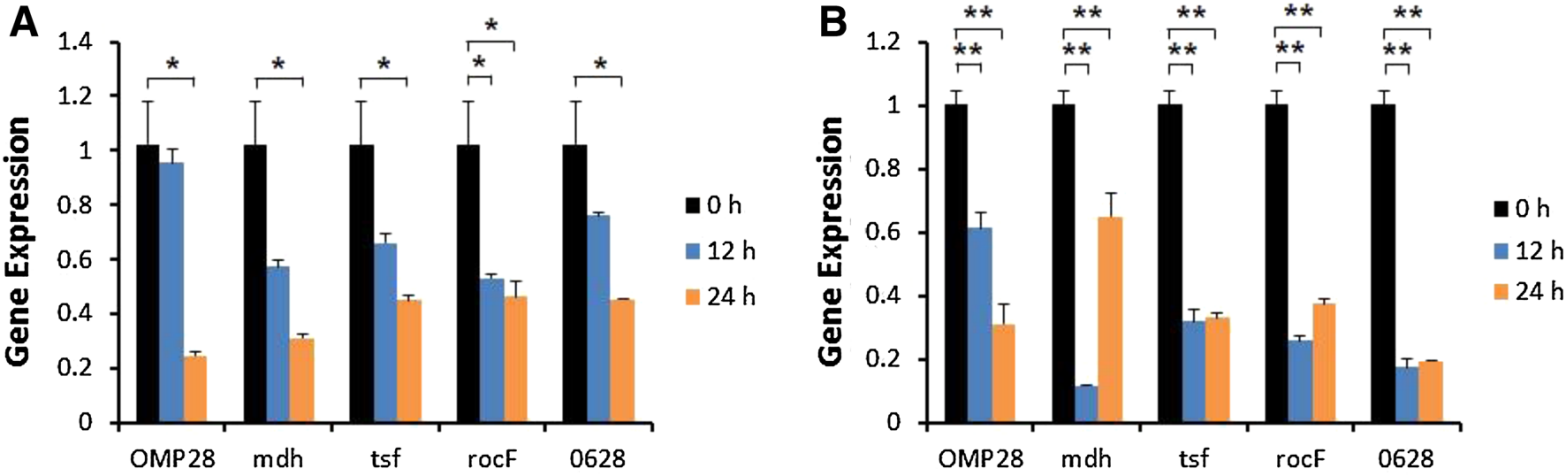
**Gene expression of interleukin-1β (IL-1β) in bovine PBMC.** Bovine PBMC were stimulated with 5 μg (**A**) and 10 μg (**B**) of five different recombinant proteins (OMP28, mdh, tsf, rocF, and 0628) of *Brucella abortus* at 0, 12, and 24 h. Gene expression was analyzed by real-time quantitative RT-PCR and normalized by the expression of β-actin.

**Figure 4:**
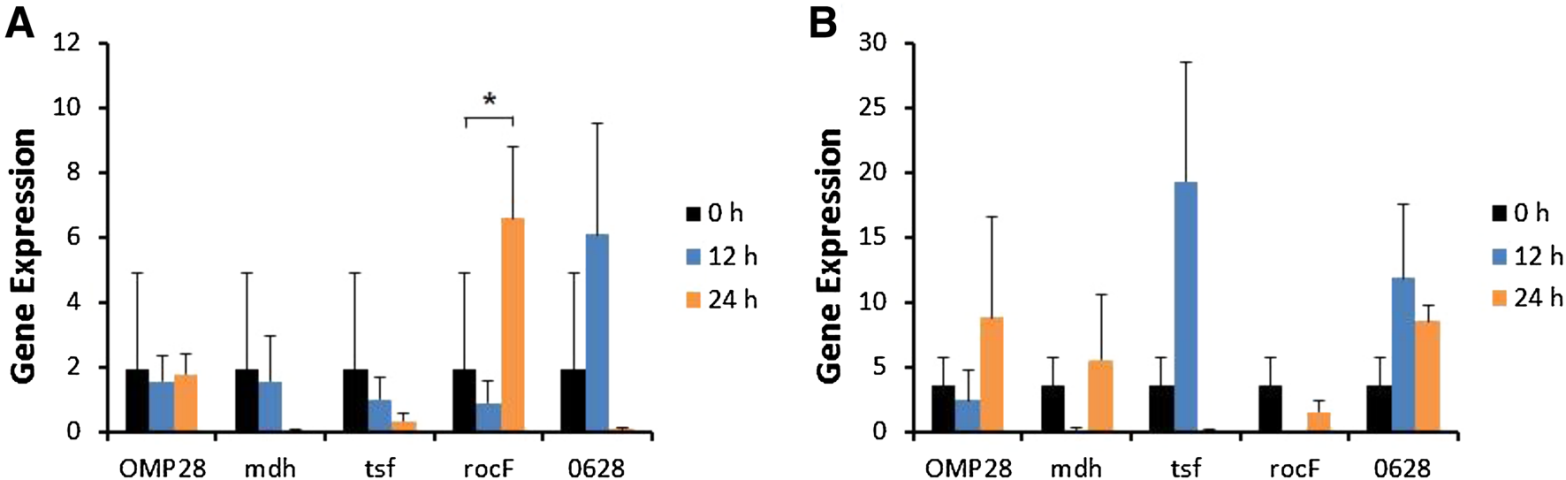
**Gene expression of interleukin-4 (IL-4) in bovine PBMC.** Bovine PBMC were stimulated with 5 μg (**A**) and 10 μg (**B**) of five different recombinant proteins (OMP28, mdh, tsf, rocF, and 0628) of *Brucella abortus* at 0, 12, and 24 h. Gene expression was analyzed by real-time quantitative RT-PCR and normalized by the expression of β-actin.

**Figure 5:**
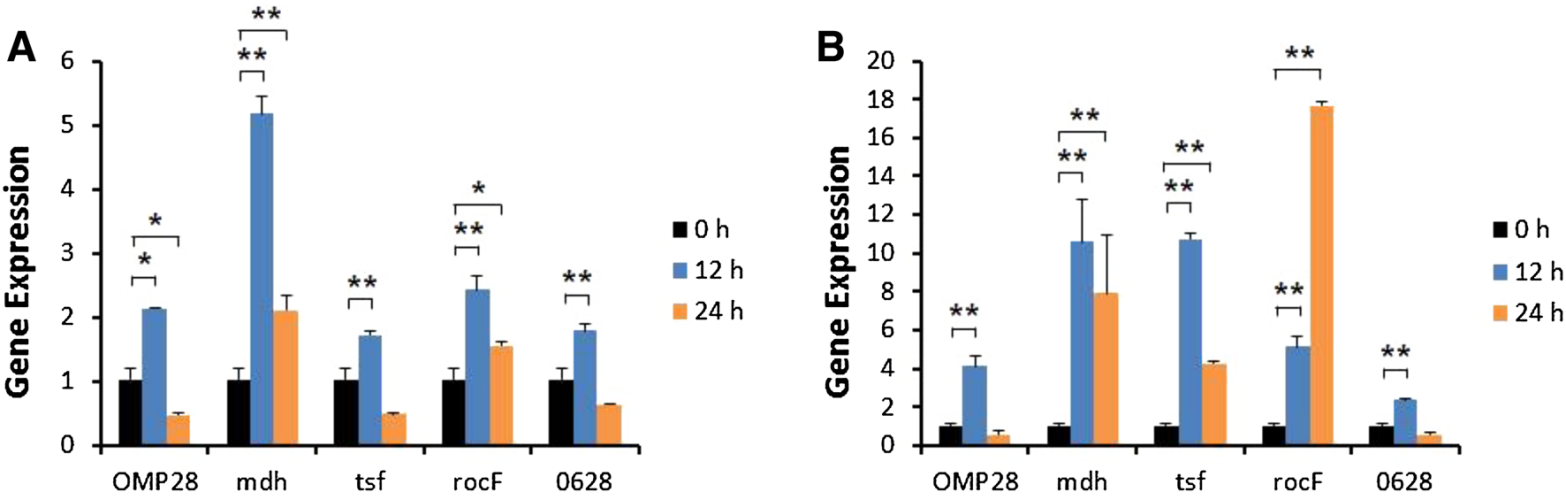
**Gene expression of interleukin-6 (IL-6) in bovine PBMC.** Bovine PBMC were stimulated with 5 μg (**A**) and 10 μg (**B**) of five different recombinant proteins (OMP28, mdh, tsf, rocF, and 0628) of *Brucella abortus* at 0, 12, and 24 h. Gene expression was analyzed by real-time quantitative RT-PCR and normalized by the expression of β-actin.

**Figure 6:**
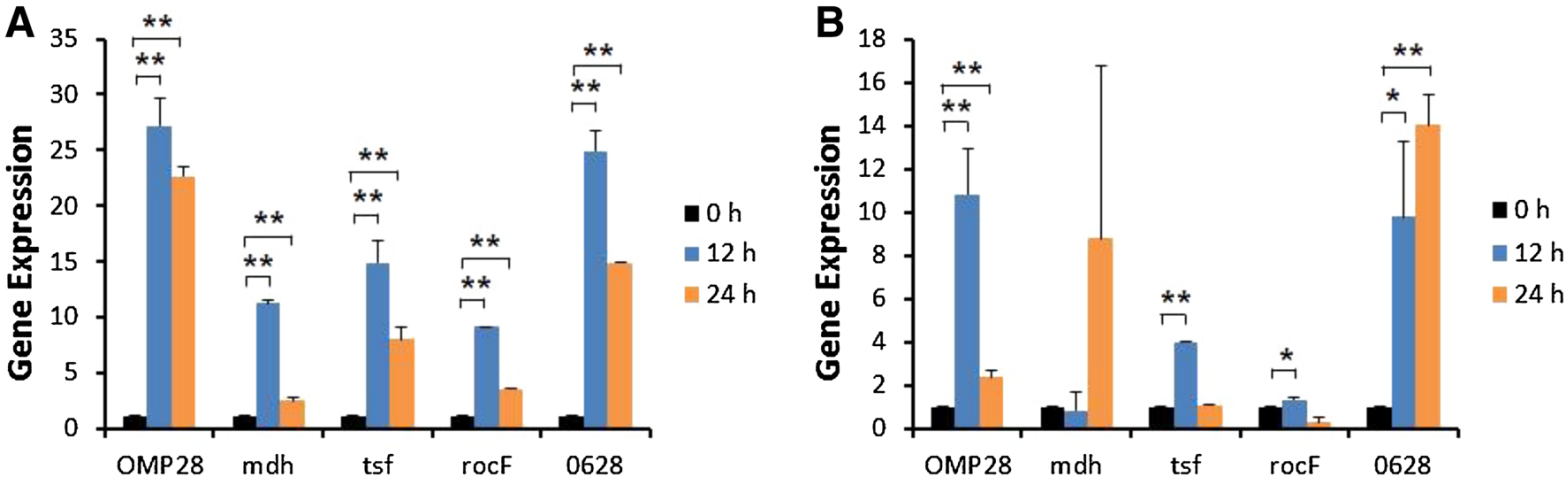
**Gene expression of interleukin-12 p40 (IL-12p40) in bovine PBMC.** Bovine PBMC were stimulated with 5 μg (**A**) and 10 μg (**B**) of five different recombinant proteins (OMP28, mdh, tsf, rocF, and 0628) of *Brucella abortus* at 0, 12, and 24 h. Gene expression was analyzed by real-time quantitative RT-PCR and normalized by the expression of β-actin.

**Figure 7:**
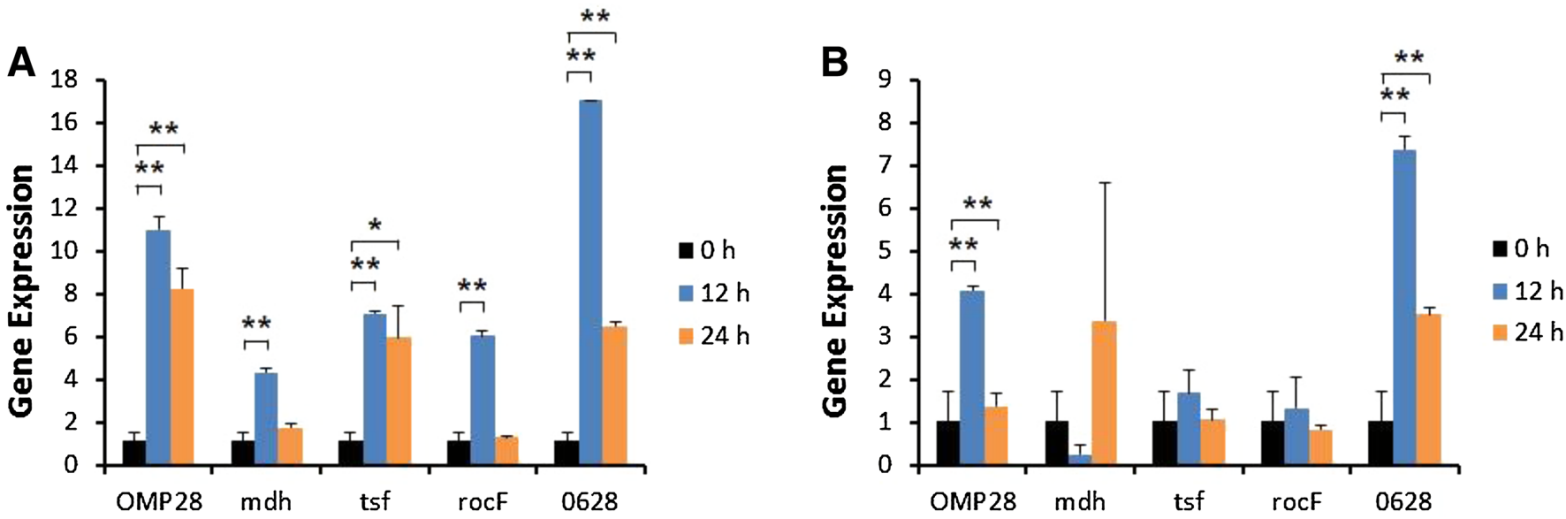
**Gene expression of interferon-γ (IFN-γ) in bovine PBMC.** Bovine PBMC were stimulated with 5 μg (**A**) and 10 μg (**B**) of five different recombinant proteins (OMP28, mdh, tsf, rocF, and 0628) of *Brucella abortus* at 0, 12, and 24 h. Gene expression was analyzed by real-time quantitative RT-PCR and normalized by the expression of β-actin.

**Figure 8:**
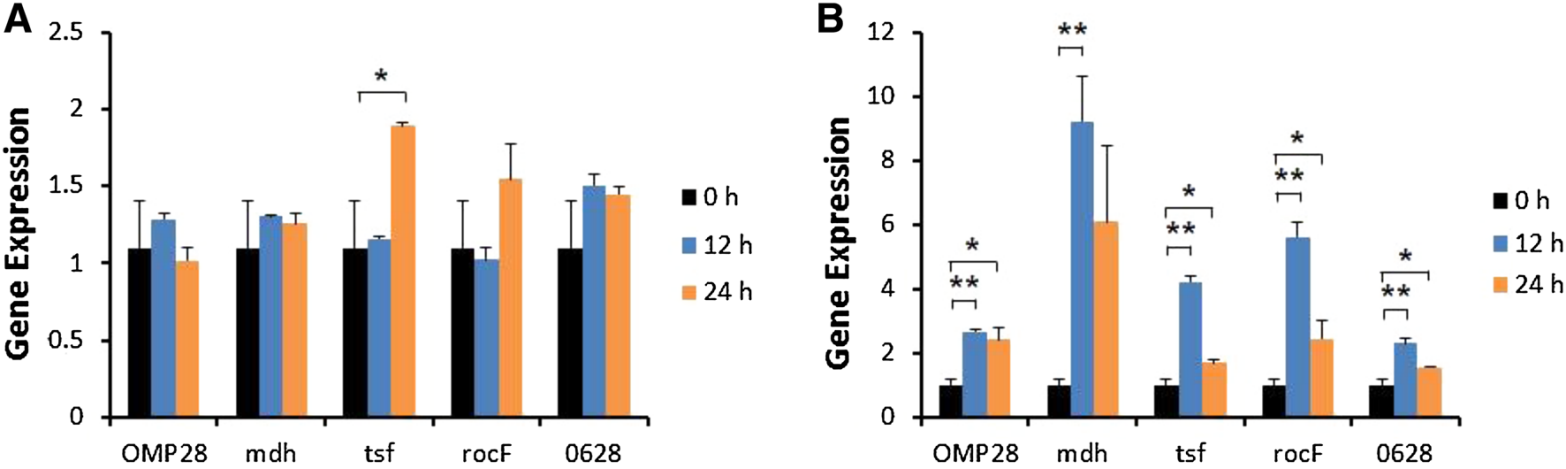
**Gene expression of tumor necrosis factor-α (TNF-α) in bovine PBMC.** Bovine PBMC were stimulated with 5 μg (**A**) and 10 μg (**B**) of five different recombinant proteins (OMP28, mdh, tsf, rocF, and 0628) of *Brucella abortus* at 0, 12, and 24 h. Gene expression was analyzed by real-time quantitative RT-PCR and normalized by the expression of β-actin.

**Figure 9:**
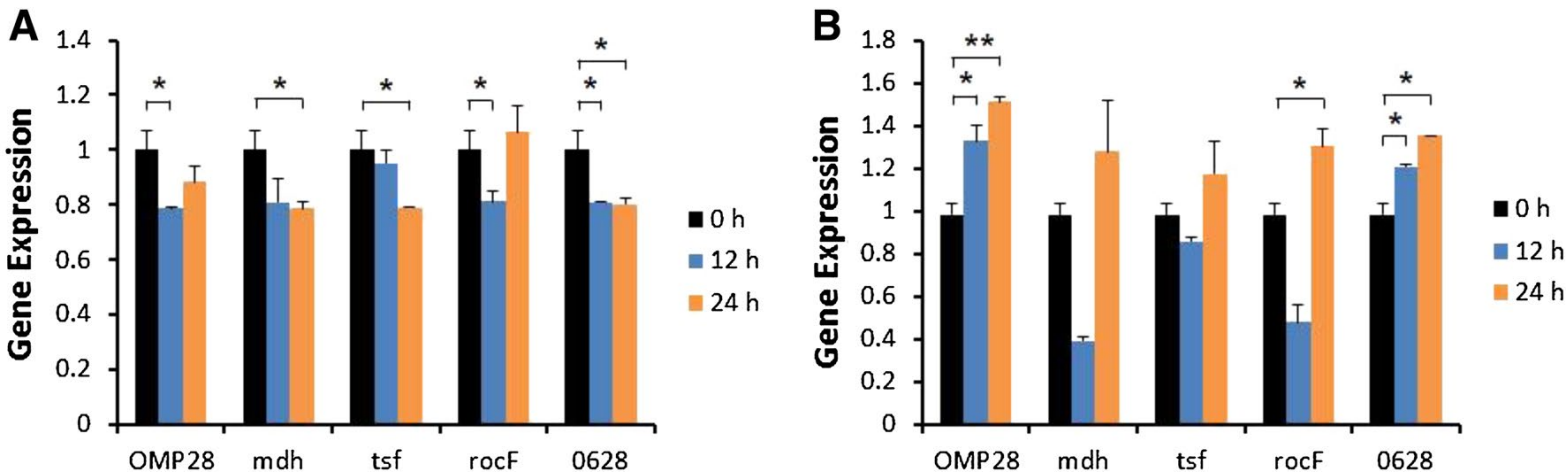
**Gene expression of Bax in bovine PBMC.** Bovine PBMC were stimulated with 5 μg (**A**) and 10 μg (**B**) of five different recombinant proteins (OMP28, mdh, tsf, rocF, and 0628) of *Brucella abortus* at 0, 12, and 24 h. Gene expression was analyzed by real-time quantitative RT-PCR and normalized by the expression of β-actin.

**Figure 10:**
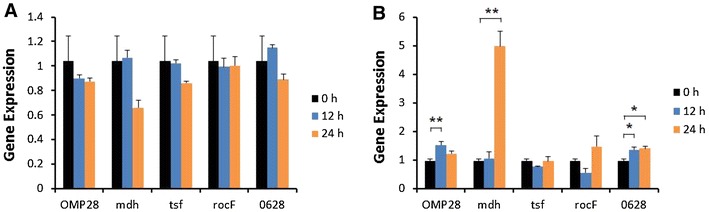
**Gene expression of Bcl-2 in bovine PBMC.** Bovine PBMC were stimulated with 5 μg (**A**) and 10 μg (**B**) of five different recombinant proteins (OMP28, mdh, tsf, rocF, and 0628) of *Brucella abortus* at 0, 12, and 24 h. Gene expression was analyzed by real-time quantitative RT-PCR and normalized by the expression of β-actin.

**Figure 11:**
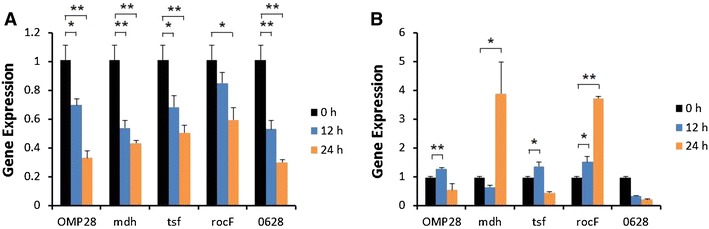
**Gene expression of Toll-like receptor 4 (TLR4) in bovine PBMC.** Bovine PBMC were stimulated with 5 μg (**A**) and 10 μg (**B**) of five different recombinant proteins (OMP28, mdh, tsf, rocF, and 0628) of *Brucella abortus* at 0, 12, and 24 h. Gene expression was analyzed by real-time quantitative RT-PCR and normalized by the expression of β-actin.

Induction of iNOS gene expression was up-regulated at 12 or 24 h after stimulation of bPBMC with 10 μg of OMP28, mdh, tsf, and rocF proteins (*p* < 0.01) while there was no significant change in the gene expression with 5 μg of all proteins (Figure 2). IL-1β gene expression was significantly down-regulated dose and time-dependently after stimulation with 5 μg and 10 μg of all proteins in the cells (*p* < 0.05, *p* < 0.01) (Figure 3). In case of IL-4, induction of significant gene expression was observed in the bPBMC stimulated with only 5 μg of rocF protein (*p* < 0.05) even though 10 μg of tsf and 0628 proteins induced the gene expression in the cells (Figure 4). Expression of IL-6 gene was highly induced time and dose-dependently with all proteins (*p* < 0.01) (Figure 5). mdh and rocF were the most effective inducers in the IL-6 gene expression in the bPBMC (*p* < 0.01). Also, 10 μg of tsf induced the higher gene expression of IL-6 at 12 h after the stimulation (*p* < 0.01). Induction of IL-12p40 gene expression was the most effective when the cells were stimulated with 5 μg of all proteins for 12 h (*p* < 0.01) (Figure 6). The induction was also effective with 10 μg of OMP28 and 0628 recombinant proteins at 12 and/or 24 h after the stimulation (*p* < 0.01). IFN-γ gene expression was significantly induced in the cells stimulated with 5 μg of all proteins for 12 h (*p* < 0.01) even though the significant induction was also observed with 10 μg of OMP28 and 0628 proteins (*p* < 0.01) (Figure 7). After stimulation of the cells with 10 μg of all proteins, TNF-α gene expression was significantly induced at 12 h and/or 24 h (*p* < 0.01). Five μg of the proteins could not induce the TNF-α gene expression except at 24 h with tsf protein (Figure 8). In apoptosis related gene expression, Bax, Bcl-2 and TLR4, the gene expression was mostly down-regulated in the cells stimulated with 5 μg of all proteins even though 10 μg of some proteins induced the gene expression at different times (Figures 9, 10, 11). The gene expression of Bax was down-regulated with 5 μg of the proteins (*p* < 0.05) while 10 μg of OMP28, rocF and 0628 proteins up-regulated the gene expression (*p* < 0.05) (Figure 9). However, the changes might not be effective even though there was significant difference. Bcl-2 gene expression was effectively up-regulated with 10 μg of mdh in 24 h stimulation (*p* < 0.01). There were no meaningful changes in the gene expression of Bcl-2 (Figure 10). TLR4 gene expression was significantly down-regulated when the cells stimulated with 5 μg of all proteins (*p* < 0.05) while 10 μg of mdh and rocF proteins induced higher gene expression of TLR4 (*p* < 0.05) (Figure 11).

In summary, the production of iNOS, IL-4 and TNF-α was not effectively induced in bPBMC by stimulation with the recombinant proteins even though some induction was observed at certain times by treatment with 10 μg of OMP28, tsf, and rocF. IL-1β gene expression was down-regulated in dose and time-dependent manner in the stimulation of cells with all proteins. IL-6, IL-12p40 and IFN-γ gene expression was effectively induced in the cells stimulated with all proteins, especially 5 μg of the proteins. The expression of a gene associated with apoptosis in bPBMC was not induced by exposure to 5 μg of the recombinant proteins. However, expression of the apoptotic genes was induced with different expression profiles at 12 or 24 h by stimulation with 10 μg of some proteins.

## Discussion

Brucellosis is a re-emerging zoonosis that has regained attention of the scientific community because pathogenesis of this disease in humans and animals has significantly evolved [3, 28]. However, the overall burden of the disease remains underestimated and has not been well studied. Eradication of brucellosis in animals is important for prevention of this disease in humans and requires optimal diagnosis along with vaccination [29]. The cellular proteins of *B. abortus* have received increased attention in the development of diagnostic techniques and vaccines given the important roles of these proteins in the early stage of infection [23]. In addition, the information will help to reveal mechanisms underlying the pathogenesis of *Brucella* infection.

*Brucella* is able to infect macrophages, and persist and replicate in the intracellular environment [30]. Identifying bacterial proteins that are necessary for intracellular survival of *Brucella* may provide new insights into mechanisms associated with pathogenesis and immune protection along with candidate antigens for diagnosis and vaccines [31–33]. Although several immunogenic proteins of *B. abortus* have been identified [24–27], roles of these proteins still remain unclear. Based on recently acquired knowledge, five different cellular proteins with unknown potential in the induction of immune responses were selected for analysis in this study and effects of the proteins on bPBMC were investigated.

To evaluate the potential of five protein antigens as diagnostic antigens, underlying mechanisms of the proteins in PBMC were investigated. Therefore, five genes were cloned, and the recombinant proteins were expressed and purified. After stimulation of bPBMC with the proteins, the expression of cytokines and apoptosis-related genes were analyzed by real-time reverse transcription-PCR. Most of the proteins induced the expression of IL-6, IL-12p40 and IFN-γ in a time- and dose-dependent manner. This result concurs with finding from a previous study showing that high amounts of IFN-γ, IL-12, and IL-6 are produced by splenocytes of mice vaccinated with chaperone protein DnaK [31]. In vivo-induced antigen technology (IVIAT) using elk (*Cervus elaphus*) revealed that mdh is a predictor of natural infection [34]. Mdh is commonly expressed during infection in cattle and elk, and is not only an immunogenic protein but also promotes bacterial pathogenesis as a new virulence factor [34, 35]. These data are similar to our results showing the effect of mdh on cytokine production.

The *B. abortus efp* mutant has slower growth in complex media and higher sensitivity to detergents [36]. The *efp* gene is also required for internalization in non-professional macrophages, HeLa cells [36]. However, the gene does not appear to be associated with virulence in professional macrophages, J774 macrophage-like cells, or mice [36].

Immune responses rely to a great extent on the recognition of foreign antigens by toll-like receptors (TLR). These receptors detect different pathogen-associated molecular patterns (PAMP), and trigger the activation of myeloid differentiation primary response gene 88 (MyD88)- and TIR-domain-containing adaptor-inducing interferon-β (TRIF)-dependent signaling pathways. This in turn leads to a wide range of cellular responses including the secretion of proinflammatory cytokines, chemokines, and type I interferons [37]. In our study, gene expression of TLR4 was down-regulated up to 24 h and up-regulated in the stimulation with the proteins. Previous studies on the TLR4 expression showed down-regulation of TLR4 expression up to 24 h and up-regulation of the expression after 24 h in the macrophage cells stimulated *B. abortus* or LPS of the bacterium [38, 39]. This phenomenon was similar with our results with bPBMC.

These results suggest that the recombinant proteins might induce proper immune responses without adverse effects (such as apoptosis) in the bPBMC. Gene expression of iNOS, TNF-α, and IL-1β was not effective in the cells by stimulation with the proteins. On the contrary, gene expression of IL-6, IL-12p40, and IFN-γ was significantly increased with the recombinant proteins. These results indicate that the adaptive immune systems are effectively activated by the proteins in bPBMC. In conclusion, the five recombinant *B. abortus* proteins examined in this study appear to induce effective adaptive immune responses in both humoral and cellular immunity without induction of inflammatory reaction.
